# Assessment of tea garden soils at An'xi County in southeast China reveals a mild threat from contamination of potentially harmful elements

**DOI:** 10.1098/rsos.180050

**Published:** 2018-08-08

**Authors:** Hai-Lei Cao, Feng-Ying Cai, Wen-Bin Jiao, Cheng Liu, Ning Zhang, Hai-Yuan Qiu, Christopher Rensing, Jian Lü

**Affiliations:** 1 Fujian Provincial Key Laboratory of Soil Environmental Health and Regulation, College of Resources and Environment, Fujian Agriculture and Forestry University, Fuzhou 350002, People's Republic of China; 2 Fujian Monitoring Center of Geological Environment, Fuzhou 350001, People's Republic of China; 3 Samara Center for Theoretical Materials Science (SCTMS), Samara State Technical University, Molodogvardeyskaya St. 244, Samara 443100, Russia

**Keywords:** tea garden soil, potentially harmful elements, ecological risk, soil quality

## Abstract

An extensive study of the spatial distribution characteristics of potentially harmful elements (PHEs) in tea (*Camellia sinensis* (L.) O. Kuntze) garden soils and ecological risk assessment at An'xi County, the birthplace of oolong tea in China, was implemented. A total of 78 soil samples were examined to determine the concentration of five PHEs (As, Cd, Cr, Hg and Pb), soil organic matter and pH by using geostatistical approaches combined with geographical information system analysis. All PHEs presented in the study area were slightly higher than their background values for provincial and national standards except Cr. Moreover, ecological risk assessment of PHEs in the tea garden soils at An'xi County was performed by means of the Håkanson method. The average ecological potential risk index (*E*_r_) of the five PHEs followed a descending order of Cd > Hg > Pb > As > Cr, and suggested a moderate ecological risk in the study area.

## 1. Introduction

Tea (*Camellia sinensis* (L.) O. Kuntze), being regarded as one of the top three beverages in the world, has become increasingly popular because of the special medicinal and healthcare functions derived from its rich organic constituents, inorganic mineral elements, and nutrient and pharmacodynamic composition [1–3]. In addition, tea plant growth effectively prevents soil erosion and provides extensive land cover and a pollution-free atmosphere, playing a key role in the maintenance of the terrestrial ecology. Over recent years, moderate application of synthetic fertilizers to tea garden soils has become common for local agricultural activities in order to supply macro- and micro-nutrients required for tea growth and to increase tea productivity [4–6]. It must not be overlooked that some trace elements, being introduced from the long-term use of fertilizers, are toxic at higher concentrations upon entering the food chain from contaminated soils, despite their foundations for metabolic activities in living organisms [7–9]. With the rapid development of human civilization, garden soil pollution has been potentially serious in some key tea-producing countries including China [10–13].

More recently, potentially harmful elements (PHEs) in soil have caused extensive, massive and regional pollution worldwide [14–16]. Higher levels of PHEs in soil affect not only plant growth but also soil biochemical processes, for example the decomposition of organic matter and nitrogen mineralization and nitrification [17–19]. Furthermore, PHEs typically accumulate in the human body due to their non-biodegradable nature and long biological half-lives for elimination. For example, a low level of Pb exposure has been known to be harmful to enzyme systems involved in blood production, while a high level of Pb exposure affects the development of intelligence in humans [20]. PHEs can be readily transferred to the human body as a consequence of dermal contact, absorption, inhalation and ingestion [21,22]. It is particularly vital for tea plantations because the accumulation and dissolution [23,24] of PHEs from tea leaves into tea infusions might be directly hazardous for humans [5,25]. Therefore, studies on the spatial distribution, source analysis and risk assessment of PHEs in tea garden soils have received unprecedented attention. Dragović *et al*. [26] employed enrichment factors to determine the relative degree of metal contamination and the anthropogenic origin of cadmium (Cd). Yan *et al*. [27] used Moran's I and geostatistical analysis to study the spatial patterns of heavy metal concentrations in soils in a mining area where Pb pollution reached the cordon and exhibited positive correlation with local plants. More recently, Karak *et al.* [28] reported the distribution and potential environmental risks posed by six heavy metals (Cd, Cr, Cu, Mn, Ni and Zn) that showed a positive correlation to inorganic and organic amendments by using a new index Tea Research Association Heavy Metal Contamination Index (TRAHMCI). Moreover, geostatistical methods enable the spatial distribution of PHEs in soil to be studied and the situations at unsampled locations to be estimated or predicted. Furthermore, the remediation needs and optimized management of tea gardens must also be taken into account when these needs are based on a viable assessment of local soil quality.

We investigated the spatial distribution characteristics of PHEs and evaluated their ecological risks at An'xi County, southeast China, where tea plantations are located in areas of higher altitude. The aims of this study were to: (i) analyse the physical and chemical characteristics of tea garden soils in the study area; (ii) determine the concentration of five PHEs (As, Cd, Cr, Hg and Pb) in tea garden soils; (iii) reveal the correlation among these PHEs and analyse their sources; and (iv) evaluate the ecological risk at An'xi County.

## 2. Material and methods

### 2.1. Description of study area

**Figure 1.**
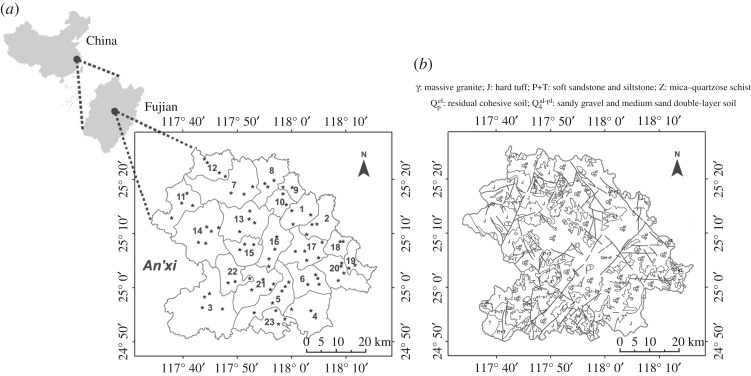
Sketch map of An'xi County with sampling spots (*a*): **1**, Hu'tou; **2**, Jin'gu; **3**, Long'juan; **4**, Long'men; **5**, Hu'qiu; **6**, Guan'qiao; **7**, Gan'de; **8**, Jian'dou; **9**, Bai'lai; **10**, Hu'shang; **11**, Fu'tian; **12**, Tao'zhou; **13**, Chang'keng; **14**, Xiang'hua; **15**, Lan'tian; **16**, Shang'qing; **17**, Peng'lai; **18**, Kui'dou; **19**, Shen'nei; 20*, Cheng'xiang and Feng'cheng; **21**, Xi'ping; **22**, Lu'tian; **23**, Da'ping; and the geological map of An'xi County (*b*). *Non-typical tea plantation towns combined as one sampling station.

An'xi County (117°36′ E ∼ 118°17′ E, 24°50′ N ∼ 25°26′ N) is the birthplace of Tieguanyin (figure 1*a*), a representative of oolong tea, located on the southeast coast of Fujian province in southeast China. An'xi County has a subtropical oceanic monsoon climate with an annual average temperature of 16–18°C and annual rainfall of around 1800 mm. The total area of 3057 km^2^ includes mountains (2269 km^2^, 74.2%), tea gardens and orchards (258.67 km^2^, 8.5%), cultivated areas (252 km^2^, 8.24%) and grassland (9.33 km^2^, 0.3%). An'xi has a history of tea production for over 1000 years and ranks first among key tea-producing counties in China. The formation and characteristics of the soils in the study area are mainly affected by topography, climate, regional hydrological conditions and parent materials (figure 1*b*). The topography of An'xi County is tilted from northwest to southeast, and its landform is mainly mountainous. The main soil types at An'xi include red soil (83.22%), yellow soil (11.95%) and small fractions of brown soil, purple soil and limestone soil. From the northwest to southeast, the distribution of soils is characterized by yellow soil and red soil. A vertical distribution of soils is observed as Latosol (0–300 m), red soil (300–880 m) and yellow soil (below 880 m). Of note, red soil is distributed largely on low hills and is one of the most observed natural soils in the study area. Moreover, a wide variety of minerals have been detected in An'xi County, including iron, kaolin, limestone, granite and geothermal minerals.

### 2.2. Soil sampling

A total of 78 surface soil samples (0–20 cm) were collected from the representative tea gardens with relatively large planting areas from 23 towns in An'xi County (figure 1*a*). Sampling sites were selected as locations that had a relatively high density of tea gardens that displayed a fairly good distribution in the study area. Samples were positioned through a mixed-point sampling method at specific sampling points under the drip line of tea trees. Samples collected from five serpentine points were mixed and then chosen by inquartation. After mixing, 2.0 kg of each soil sample was stored in self-sealing plastic bags and sent back to the laboratory and dried naturally. Plant roots and visible foreign matter were removed manually from the samples, which were ground in an agate mortar, followed by passing through 20 and 100 mesh nylon sieves, respectively, for further testing. The authors confirm no permission to carry out fieldwork was required.

### 2.3. Physical and chemical analysis

Soil pH (±0.1) was measured in a 1 : 5 soil-to-water suspension after stirring for 2 h. Soil organic matter was determined by a potassium dichromate volumetric and external heating method via mass loss on ignition at 600°C. The concentrations of Cd and Pb were determined by graphite furnace atomic absorption spectrometry on a Koromee KAAS-2001E spectrometer (Shanghai, China). As and Hg contents were measured by atomic fluorescence spectrometry on a Jitian AFS-933 spectrometer (Beijing, China). The concentration of Cr was measured on a flame atomic absorption spectrophotometer (INESA 361MC, Shanghai, China). Calibration was performed on 10 sets of samples using internal standards via the standard curve approach. Special care was taken with preparing and analysing samples to minimize contamination from air, glassware and reagents. Controlled measurements on internal reference materials, reagent blanks and duplicated soil samples selected randomly from the set of available samples were applied to assess contamination precisely. The precision, accuracy and detection limits of the PHE tests are listed in table 1.

**Table 1.** The precision, accuracy and detection limit of PHEs tested in this study.

PHE	detection limit (mg kg^–1^)	relative error (%)	relative deviation (%)
Cd	0.01	≤4	≤9
Pb	0.1	≤1.5	≤9
Hg	0.002	≤5	≤12
As	0.01	≤5	≤7
Cr	5	≤5	≤3

### 2.4. Data analysis

Data analyses such as descriptive statistics and correlation analysis were performed by SPSS 20.0 software and applied to 66 statistical sampling points (after removing abnormal points) [29]. Geostatistical analysis was used to generate visual images of spatial heavy metal distribution by using ArcGIS 10.2 [30]. Geostatistical methods are very important in the study of variations of soil spatial properties and regional heavy metal pollution. They have been proved to be among the most effective methods of analysing the spatial distribution variability of heavy metals in soil. Geostatistical spatial analyses were used to generate a semi-variance map and describe spatial variability. ArcGIS 10.2 was extensively employed, including the semi-variogram of the calculation and fitting comparison, Kriging spatial interpolation (ordinary Kriging) and simulation error analysis, etc. [31].

Pearson's correlation analysis of soil parameters generally provides important information for identifying the relationship between the variables and PHE sources. Thus, a multivariate statistical analysis was conducted using SPSS 20.0. Based on the above results, principal component analysis (PCA) was employed to confirm the potential tracers of PHEs, natural enrichment or anthropogenic inputs. Moreover, varimax rotation was employed to minimize the spatial dimension of variables. The original data on the PHEs were normalized by logarithm and Box–Cox transformation before geostatistical analysis, and then the processed data were analysed through the ArcGIS 10.2. Kriging interpolation and GIS mapping were applied to establish the spatial distribution maps of PHEs [32].

### 2.5. Ecological risk assessment method of PHEs in soil

Potential ecological risk assessment of PHEs in tea garden soils at An'xi County was assessed using the Håkanson method [33]. The average ecological potential risk index (*E*_r_) of PHEs was calculated as follows:

2.1 $$\text{RI}=\Sigma E_{r,i},$$

2.2 $$E_{r,i}=T_{r,i}\times C_{f,i}$$

2.3 $$\;\text{and}\;C_{f,i}=\frac{C_{D,i}}{C_{R,i}},$$

where *E*_r,i_ is the potential ecological risk index of an individual element; *T*_r,i_ is the toxicity coefficient of an individual PHE (10 for As, 30 for Cd, 2.0 for Cr, 40 for Hg and 5.0 for Pb) [34]; *C*_f,i_ is the single factor pollution index of an individual PHE; *C*_D,i_ is the actual content of PHEs in soil; and *C*_R,i_ is the reference values of PHEs [35,36].

## 3. Results and discussion

### 3.1. Physical and chemical parameters of soils in An'xi County

**Figure 2.**
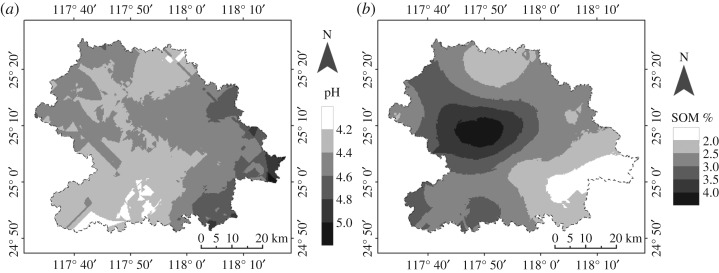
Spatial patterns of soil pH (*a*) and SOM (*b*) at An'xi County.

The main physical and chemical parameters including pH and soil organic matter (SOM) were determined for tea garden soils at An'xi County, which showed general features as follows: (i) wide soil pH range; (ii) low SOM contents with large variation; and (iii) soil degradation and decline of soil fertility. Moreover, the use of chemical fertilizers, root exudation and biological cycling of tea plants will cause soil acidification that is favourable for tea growth in acidic conditions. However, recent changes in land use and soil amendment, especially the excessive addition of dolomitic lime, have resulted in the appearance of less favourable soils. Soil pH in the study area varies from 3.6 to 5.65 with an average value of 4.45 (table 2 and figure 2*a*), among which about 85.5% of soils are in the pH range of 4.0–5.5. The small fraction of soils (pH < 4.0 or pH > 6.5; *ca* 11.8%) that are less favourable for tea growth are mainly located in the northeast and northwest fringes (pH < 4.0; **8** Jian'dou, **9** Bai'lai, **10** Hu'shang and **11** Fu'tian) and eastern (pH > 6.5; **2** Jin'gu and **19** Shen'nei) regions of An'xi County, and are most likely affected by local human activities and communication with the neighbouring areas.

**Table 2.** Concentrations of PHEs (mg kg^–1^) and reference background values of provincial and national standards [35,36], pH and SOM in tea garden soils at An'xi County (s.d., standard deviation; CV, coefficient of variation; number of statistical samples *N* = 66).

	An'xi County								background value		
element	min	max	mean	s.d.	CV (%)	skewness	kurtosis	K–S	provincial^a^	national^b^	CERT^c^
As (mg kg^–1^)	1.25	18.10	5.50	3.34	60.73	1.39	2.41	0.126	5.78	26	40
Cd (mg kg^–1^)	0.01	022	0.09	0.06	66.7	0.33	–0.95	0.053	0.05	0.09	0.30
Cr (mg kg^–1^)	2.50	46	11.98	8.44	70.45	1.85	4.57	0.110	41.4	65	150
Hg (mg kg^–1^)	0.01	0.35	0.10	0.06	60.0	1.87	5.05	0.012	0.08	0.04	0.30
Pb (mg kg^–1^)	5.50	117.0	43.42	27.20	62.64	1.14	0.86	0.058	34.9	23	250
pH	3.60	5.65	4.45	0.46	10.3	0.81	0.42	0.423	—	—	—
SOM (%)	0.8	6.2	2.83	11.27	44.75	0.55	–0.02	0.541	—	—	—

^a^Soil background value of Fujian province [35].

^b^Soil background value of China [36].

^c^Quality requirements of the soil environment in Chinese tea gardens (NY/T 853-2004).

Although SOM corresponds to only a small fraction of soil constituents, it is significantly beneficial for soil formation, fertility, sustainable development of agroforestry and environmental protection [37]. In general, SOM in the study area ranged from 0.8% to 6.2% with an average value of 2.75% (table 2 and figure 2*b*). Based on the SOM contents, soil impoverishment can be classified as follows: severely poor (SOM < 1), poor (1.0 < SOM < 1.5), mild marginal (1.5 < SOM < 2.0) and rich (SOM > 2.0). Thus, most areas in An'xi County were classified as rich and two small regions, Gan'de (**7**; figures 1 and 2*b*) and Guan'qiao/Shen'nei/Cheng'xiang (**6**/**19**/**20**; figures 1 and 2*b*), as mild marginal. These results indicate that the use of organic fertilizers is only recommended in some local areas rather than in a broad region.

### 3.2. Statistical analysis of PHEs in tea garden soils at An'xi County

#### 3.2.1. Descriptive statistics

Basic statistics for concentrations of PHEs in the top soil samples are shown in table 2. The mean concentration of Cr in the study area was significantly lower than the local background value, and the mean concentration of As at An'xi was slightly lower than the average background value [36]. However, the average concentrations for Cd, Hg and Pb were higher than their background values by up to 1.80, 1.25 and 1.24 times, respectively. The comparative medium coefficient of variation (CV) for all PHEs showed a fairly large geochemical variability in concentrations for each of two metals in the sampled soils. Generally, the degree of variation can be divided into three levels: CV < 10% is considered as weak variability, 10% < CV < 100% defines moderate variability, and CV > 100% is strongly variable [38,39]. The CV of all PHEs at An'xi was moderate variability, following an order of Cr > Cd > Pb > As > Hg, which indicated the concentration of elements showing moderate geochemical variation. The Kolmogorov–Smirnov test indicated that concentrations of As, Cd, Cr and Pb in soils were in compliance with normal distribution, while Hg showed abnormal distribution. However, the Hg concentration passed the normality test after logarithmic transformation. The skewness, being a parameter related to the normal distribution, also confirmed the above results. Furthermore, the kurtosis value suggested that Hg distribution had a distinct cluster around relatively low values. According to the environmental requirements for growing areas of tea in China (NY/T 853-2004), the As, Cd, Cr and Pb concentrations in all soil samples were below the threshold concentration, while the Hg concentration exceeded the guideline value in less than 2% of the samples. Therefore, the soils at An'xi County were slightly contaminated with Hg, and a small amount of accumulated Cd and Pb, which was also generally the case for soils in other agricultural areas in China.

#### 3.2.2. Correlation analysis

**Figure 3.**
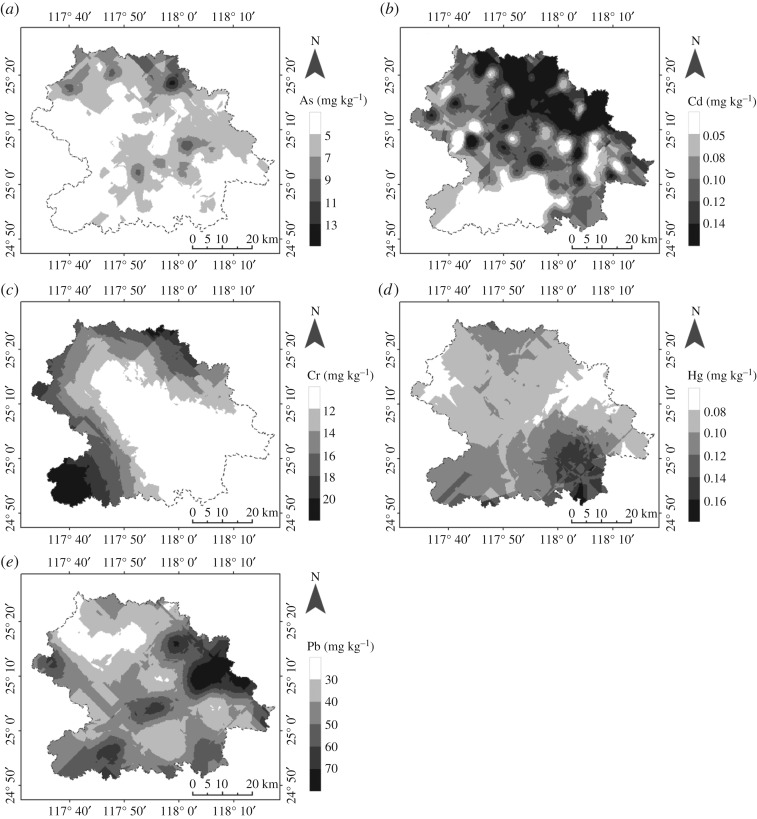
(*a*–*e*) Spatial patterns of As, Cd, Cr, Hg and Pb in soils from An'xi County.

Pearson correlation coefficients [40,41] were performed to analyse the correlation between variables, including SOM, pH and PHEs (table 3). The pH showed significant positive correlation with Cd (*r* = 0.325a) and Pb (*r* = 0.353a), which suggested that Cd and Pb have a tendency to accumulate with increasing pH, as indicated in figures 2*a* and 3*b,e*. Of special note, significant positive correlations were found between the elemental pairs Cd–Pb (*r* = 0.296b) at the 0.05 significance level, implying their same sources and similar pathways of accumulation.

**Table 3.** Correlation coefficients of the concentrations of PHEs.

	SOM	pH	As	Cd	Cr	Hg	Pb
SOM	1.000	–0.033	0.046	–0.04	0.169	0.065	0.029
pH		1.000	–0.055	0.325^a^	0.124	0.183	0.353^a^
As			1.000	0.107	0.111	0.161	0.108
Cd				1.000	0.112	0.068	0.296^b^
Cr					1.000	0.068	0.063
Hg						1.000	0.041
Pb							1.000

^a^Correlation is significant at *p* < 0.01 (two-tailed)

^b^Correlation is significant at *p* < 0.05 (two-tailed).

#### 3.2.3. Principal component analysis

PCA has been widely applied to study the sources of PHEs in soil [42]. The PCA results suggested that eigenvalues of three components were greater than 1.0 and accounted for *ca* 70.1% of the data variation. It was observed from the rotated component matrix that the first principal component (PC1) was closely related to Cd and Pb, and explained *ca* 26.1% of the total variation (table 4). The second principal component (PC2) included Hg and As, whereas Cr was classified as the third principal component (PC3). PC2 and PC3 contributed *ca* 22.1% and *ca* 21.9% of the total variation, respectively.

**Table 4.** PHE communality of a rotated component matrix for As, Cd, Cr, Hg, Pb in soils from An'xi County. Extract method: principal component analysis; rotation methods: orthogonal rotation under Kaiser standard.

	component		
PHE	PC1	PC2	PC3
As	0.160	0.679	0.156
Cd	0.783	0.036	0.127
Cr	0.048	0.070	0.981
Hg	–0.056	0.831	–0.062
Pb	0.812	0.065	–0.054

##### 3.2.3.1. Component 1: Cd/Pb

Cd and Pb showed similar distribution patterns in the soil sample profiles, featured by massive enrichment in the east An'xi area (figure 3*b,e*), especially in high pH areas (**2**, **19** and **4**; figure 1). The percentage of Cd and Pb over background values was 68% and 61.8%, respectively. These elements are mainly derived from anthropogenic sources that were associated with agricultural and industrial activities.

##### 3.2.3.2. Component 2: As/Hg

The percentage of As and Hg (figure 3*a*,*d*) over background values was 36.0% and 58.4%, respectively. The main source of these heavy metal pollutants was more likely to be waste produced by various industrial activities that entered the soils through atmospheric deposition, surface run-off and sewage irrigation.

##### 3.2.3.3. Component 3: Cr

Cr could be defined as a natural resource, as the average concentrations of Cr are well below the background values. The abnormal distribution of Cr in soils was mainly related to Permian and Jurassic geosphere in which Cr content can be low. Moreover, the Cr content might be affected by iron and lime ores, as well as coal mines, and thus we conclude that the contents and spatial distribution of Cr were mainly controlled by natural factors [43], such as parent material and pedogenic processes.

### 3.3. Geostatistical analysis of PHEs in tea garden soils at An'xi County

The geostatistical method has been proven to be one of the most effective methods of analysing the spatial distribution of PHEs in soil and discovering regional PHE pollution. Firstly, based on the comparison and cross-validation of fitting parameters, we chose suitable kriging interpolation models for the heavy metals in soil, and the results are presented in table 5. The best-fitting theoretical models for the semi-variograms of As, Cd, Cr and Pb were the Gaussian model, whereas for Hg it was the spherical model. Then kriging interpolation was performed on all the samples as a digital mapping method to obtain visual information on the spatial distributions of heavy metals in soil.

**Table 5.** Best-fitting semi-variogram models of heavy metals in soil and their parameters (M, mean; RMS, root mean square; ASE, average standard error; MS, standard means; RMSS, standard root mean square)

		cross-validation				
element	model	M	RMS	ASE	MS	RMSS
As	Gaussian	–0.0008	3.295	3.167	–0.0006	1.0368
Cd	Gaussian	–0.002	0.076	0.08	–0.0245	0.9661
Cr	Gaussian	–0.1151	8.416	7.16	–0.0082	1.1558
Hg	spherical	–0.0003	0.061	0.062	–0.0025	0.9795
Pb	Gaussian	–0.1182	26.748	27.046	–0.0016	0.9801
pH	exponential	–0.0162	0.479	0.442	–0.0329	1.0785
SOM	Gaussian	0.0698	11.720	11.299	0.0079	1.0406
IR	Gaussian	–1.3909	54.804	54.388	–0.0228	1.0069

Spatial distribution maps of As, Cd, Cr, Hg and Pb in An'xi County are presented in figure 3. The distribution of As and Cd content (figure 3*a*,*b*) exhibited similar characteristics with a general decreasing tendency from northeast to southwest, while Hg and Pb (figure 3*d*,*e*) displayed opposite spatial patterns compared with those of As and Cd, by increasing from northeast to southwest. The Cr content (figure 3*c*) showed a uniform distribution in the whole area, with a noticeable enrichment at the border of Hu'tou (**1**), Shang'qing (**16**) and Peng'lai (**17**).

**Figure 4.**
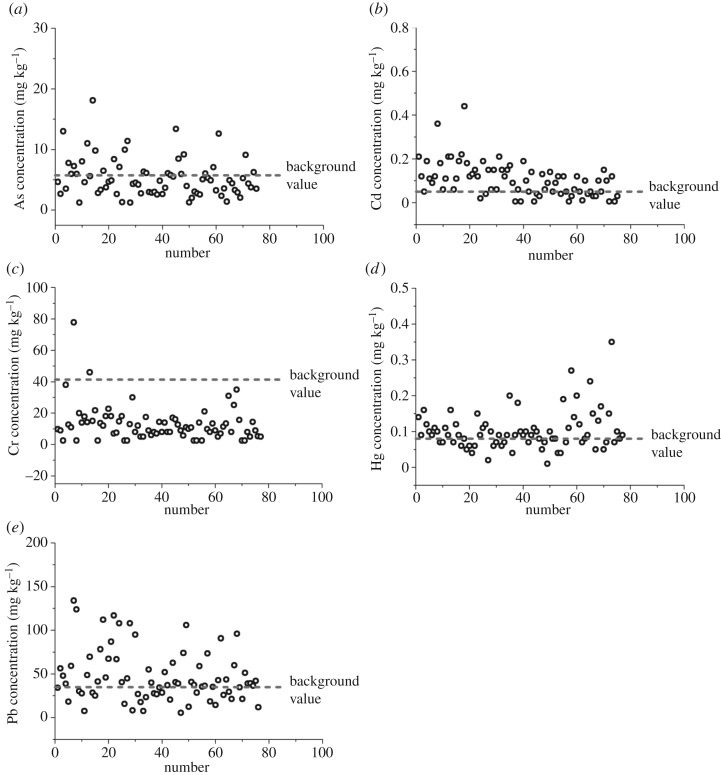
Concentrations of PHEs in tea garden soils in An'xi County and provincial background values.

Concentrations of As, Cd, Cr, Hg and Pb in soils from An'xi County, together with soil background values of PHEs at provincial and national standards, are presented in table 2. Metal concentrations of As, Cd, Cr, Hg and Pb were in the range of 1.25–18.1, 0.01–0.22, 2.5–46, 0.01–0.35 and 5.5–117 mg kg^–1^, with mean values (mean ± s.d.) of 5.50 ± 3.34, 0.09 ± 0.06, 11.98 ± 8.44, 0.10 ± 0.06 and 43.42 ± 27.20 mg kg^–1^, respectively. The mean concentration of PHEs in soil followed a descending order of Pb > Cr > As > Cd > Hg. In comparison with the provincial and national standards, the average As and Cr concentrations (table 2 and figure 4*a*,*c*) were well below the background values, whereas the Cd, Hg and Pb concentrations (table 2 and figure 4*b*,*d*,*e*) were slightly higher than their background values (no more than three times), while they were considerably lower than those in the neighbouring areas [44]. The calculated standard-reaching rates for all five PHEs were 100% (As), 100% (Cd), 100% (Cr), 98.7% (Hg) and 100% (Pb), suggesting that the soil quality in most of the study area satisfied the national standard of the Environmental Requirement for Growing Area of Tea (NY-T 853-2004) in China.

### 3.4. Potential ecological risk assessment of soils in An'xi County

**Figure 5.**
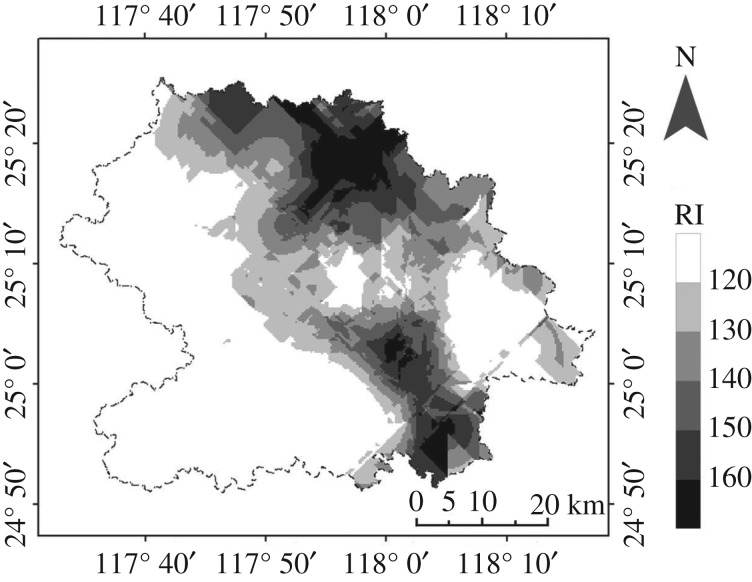
The spatial pattern of ecological potential risk indices at An'xi County.

The potential ecological risk index (*E*_r_) proposed by Håkanson [33] has been used to classify the ecological risk upon exposure of the five PHEs in the study area. The *E*_r_ for As, Cr and Pb fitted well into the catalogue of slight ecological risk, whereas the *E*_r_ for Cd and Hg was distributed widely among various groups of ecological risk, with a strong/extremely strong ecological risk of 25.76/29.79% (for Cd) and 12.12/46.97% (for Hg). The average potential ecological risk index of PHEs decreased in the order Cd > Hg > Pb > As > Cr, in which the levels of Cd and Hg contamination reached moderate ecological risk that indicated improvements to local soil quality were required [45]. The integrated RI of 125.24 also indicated that the tea garden soils at An'xi County were exposed to moderate risk of PHE contamination (figure 5).

### 3.5. Source analysis of soil PHEs at An'xi County

As discussed above, the Cd and Hg contents have shown a medium level of ecological risk in the study area by exceeding the provincial background values by 55.6% and 59.1%, respectively (figure 4*b*,*d*). According to the spatial distribution maps (figure 3*b*,*d*), Cd and/or Hg accumulation was observed in most areas of An'xi County. To be specific, Cd pollution was mainly in the form of non-point sources in the soil, whereas Hg entered the soil mainly as point sources. Cd was the characteristic element in soils when extensive agricultural activities were applied [46,47]. This is indeed the case in the study area where tea plantation is mainly engaged. Thus, it could be rationalized that the vast majority of non-point Cd pollution came from the usage of pesticides and fertilizers during tea growth. The Hg content in soils had a positive correlation with the distance to the power plants located at Long'juan and Long'men (**3** and **4**; figure 1). The production of flue gas by the power plants might be responsible for Hg accumulation in and around areas **3** and **4**.

Despite As, Cr and Pb being classified as slight ecological risk in the study area, Pb contents in *ca* 59.0% of the sampled areas exceeded the provincial background value (figure 4*e*), which was possibly related to the nearby Pb–Zn mine in the north [48]. On the other hand, the even higher Pb contents in those areas could also partially be associated with vehicular exhausts originated from the use of leaded gasoline and deterioration of mechanical parts, e.g. wear and tear of tyres [41,49]. The Cr content showed a low ecological risk and percentage of counting over the background value (figure 4*c*). The As content displayed both low accumulation and low ecological risk in the study area (figures 3*a* and 4*a*).

Overall, the region-dependent characteristics of PHEs in tea garden soils were correlated with multiple factors, i.e. industry, agriculture and the transportation network. Moreover, the average concentrations of PHEs in different tea plantations are listed in table 6. Individual PHE pollution did appear in very few tea plantations in local areas. These data showed that the appearance of a maximum PHE is independent of other elements [24,50,52,54]. Of special note, Hg levels in all tea plantations were similar, while the referenced Cd level in Indea appeared to be noticeably high [24]. Therefore, it is advisable to regularly monitor the local soil environment for the sake of pollution control in tea plantations.

**Table 6.** Average As, Cd, Cr, Hg and Pb concentrations (mg kg^–1^) in tea garden soils in the study and reference areas.

site		As	Cd	Cr	Hg	Pb	located area	reference
China	Fujian	5.50	0.09	11.98	0.10	43.42^a^	*117°36′ E ∼ 118°17′ E, 24°50′ N ∼ 25°26′ N*	this study
	Yangtze delta	22.9^a^	0.08	n.a.	n.a.	n.a.	*111°05′ E ∼ 123°E, 27°50′ N ∼ 35°20′ N*	[50]
	Yunnan	21.2	0.23	48.2	0.10	41.4	*99°56′ E ∼ 101°50′ E, 21°08′ N ∼ 22°36′ N*	[51]
	Hunan	12.4	0.07	92.2^a^	0.074	42.7	*111°53′ E ∼ 114°15′ E, 27°51′ N ∼ 28°41′ N*	[52]
	Guizhou	7.99	0.10	20.85	0.045	36.6	*106°07′ E ∼ 107°07′ E, 26°11′ N ∼ 27°22′ N*	[53]
	Guizhou	7.7	0.16	43.6	0.19^a^	37.8	*106°59′ E ∼ 107°22′ E, 26°05′ N ∼ 26°4′ N*	[54]
	Sichuan	6.14	0.069	16.2	0.054	7.63	*105°27′ E ∼ 107°58′ E, 30°01′ N ∼ 32°45′ N*	[55]
	Sichuan	5.42	0.28	19.0	0.049	42.4	*102°15′ E ∼ 104°15′ E, 28°28′ N ∼ 29°56′ N*	[53]
	Shaanxi	n.a.	0.14	21.5	n.a.	33.3	*106°21′ E ∼ 106°57′ E, 32°53′ N ∼ 33°38′ N*	[56]
Turkey		n.a.	0.2	n.a.	n.a.	29.83	Northeast Turkey	[11]
India		n.a.	11.2^a^	1.74	n.a.	23.8	South India	[24]

^a^Maximum value.

## 4. Conclusion

In this study, we presented the spatial distribution of PHEs in tea garden soils, as well as a rational ecological risk assessment for An'xi County, southeast China. A vast majority of the sampled soils met the standards as regulated by the China Environmental and Technical Requirements for Tea Production (NY/T 853-2004) in the study area. The coefficients of variation of PHEs in the study area followed the order of Cr > Cd > Pb > As > Hg. The average potential ecological risk index (*E*_r_) of PHEs in the study area decreased in the order Cd > Hg > As > Pb > Cr, in which the levels of Cd and Hg contamination reached moderate ecological risk and indicated that an improvement in the local soil quality was required. The integrated RI of 149.3 also indicated that the tea garden soils at An'xi County were exposed to a moderate risk of PHE contamination. Moreover, all five PHEs displayed satisfactory standard-reaching rates in the study area, which revealed a mild threat from PHE contamination in tea garden soils at An'xi County.

## Data accessibility

Original data have been deposited to the Dryad Digital Repository, http://dx.doi.org/10.5061/dryad.5f6v8 [57].
